# *Morchella esculenta* polysaccharide attenuate obesity, inflammation and modulate gut microbiota

**DOI:** 10.1186/s13568-022-01451-5

**Published:** 2022-09-03

**Authors:** Ata Ur Rehman, Asif Iqbal Khan, Yi Xin, Wang Liang

**Affiliations:** 1 Department of Biotechnology, College of Basic Medical Science, Dalian Medical University, Dalian, 116044 China; 2 Clinical Stem cell Research Centre, First Affiliated Hospital, Dalian Medical University, Dalian, 116044 China

**Keywords:** *Morchella esculenta*, Polysaccharide, Obesity, Gut microbiota, Inflammation

## Abstract

Edible mushrooms have now been suggested as promising sources of biological functional ingredients and are the subject of the most recent nutrition research and novel functional foods. Polysaccharides from mushrooms exhibit impressive biological effects, notably against obesity. Obesity is a chronic metabolic disorder characterized by chronic inflammation, gut dysbiosis, and hyperpermeability of the colon. Here, we prove that mushrooms *Morchella esculenta* polysaccharide (MEP) effects on HFD-induced obesity, colonic inflammation, and gut microbiota dysbiosis. Our findings demonstrate MEP supplementation attenuates obesity parameters and reduces inflammation in the colon via regulation of Toll-like receptor 4 (TLR4), nitric oxide synthase (iNOS), cyclooxygenase-2 (COX-2), and inactivation of nuclear factor kappa B (NF-κB). Furthermore, MEP administration restores gut microbiota dysregulation by ameliorating *Firmicutes* to *Bacteroidetes* proportion as well as enhancing beneficial bacteria, like *Lactobacillus*, and inhibiting pathogenic bacteria like *Enterococcus.* MEP improves gut integrity by increasing tight junction proteins (TJs) and reducing endotoxin levels by controlling Lipopolysaccharide (LPS) in HFD-induced obese mice. These results demonstrated the therapeutic efficacy of MEP in attenuating HFD-induced obesity via regulating inflammatory cascades, ameliorating the gut microbiome, and modulating gut integrity.

**Supplementary Information:**

The online version contains supplementary material available at 10.1186/s13568-022-01451-5.

## Introduction

Mushrooms have been consumed and cultivated worldwide for hundreds of years due to their enormous biological activity, dietary importance, and delicious taste. The most widely known edible mushrooms species are *Lentinula edodes, Agaricus bisporus, Auricularia auricular, Flammulina velutipes, Ganoderma lucidum, Pleurotus eryngii* (Kozarski et al. 2020; Tung et al. 2020). Edible mushrooms have now been suggested as promising sources of biological functional ingredients and are the subject of the most recent nutrition research, in addition to expanded uses in innovative functional foods (Blumfield et al. 2020). A wide range of nutraceutical substances, including polysaccharides, exhibit impressive biological effects, notably against obesity (Mingyi et al. 2019).

Obesity is the world’s biggest health issue, which has been related to various health ailments, shortened life expectancy, and has reached epidemic levels in developing nations (Bray et al. 2016). Nearly 38.5% of men and 39.4% of women were reported to be overweight, with 10.1% of men and 14.8% of women obese (Chooi et al. 2019). If current trends persist, it is predicted that by 2025, 1 billion adults (almost 20% of the world population) would be obese (Ezzati et al. 2017). Obesity predisposes people to develop and provoke a wide variety of diseases, including chronic low-grade inflammation, fatty liver disease, type 2 diabetes, insulin tolerance, respiratory problems, gastrointestinal issues, coronary disease, and multiple forms of cancer (Segula 2014).

Nowadays, it is commonly known that metabolic syndrome is linked with chronic low-grade inflammation, although the molecular mechanism of inflammation is unclear (Hotamisligil 2006). A plethora of research indicates that the hyper-permeable gut is a typical feature found in obese individuals and animals (Ahmad et al. 2017). A hyper-permeable gut ultimately induces systemic or local inflammation owing to enhanced antigen susceptibility, contributing to compromised gut barrier function, decreased expression of tight junction proteins (TJPs), and increased gut permeability to opportunistic pathogenic microbes (Brun et al. 2007). In comparison, high-fat diet-induced obesity increased the levels of LPS in the blood, eventually leading to inflammation and insulin resistance. LPS is one of the key constituents of gram-negative bacteria’s outer membrane that activates several inflammation-related transcription factors, resulting in gut barrier destruction (Baranova et al. 2016).

The role played by the intestinal microbiome in the development of obesity and associated diseases are well known (Liou et al. 2013). The gut microbiota regulates host physiology by utilizing nutrition derived from diverse and indigestible food compounds such as polyphenols and polysaccharides (Etxeberria et al. 2013). The perturbation of microbial composition contributes to a condition called intestinal dysbiosis (Belizario and Faintuch 2018). Multiple gut microbiota dysbiosis has also been linked to a wide range of human diseases, including obesity, inflammatory diseases, neurological and metabolic disorders (Kaur et al. 2011; Tilg and Moschen 2014). Several in-vivo investigations have shown that intestinal dysbiosis is an important contributor to type 2 diabetes (T2DM) and obesity (Daniel et al. 2014; Turnbaugh et al. 2008). Other research in the obesity model indicates that obesity-induced gut dysbiosis caused by genetic or environmental factors damages gut integrity (Cani et al. 2008, 2009). Thus, the dynamic change in gut microbiota composition via high-fat enriched diet intake not only induces obesity and inflammation but also damages gut integrity.

In recent years, numerous medications have been developed to treat obesity and health problems linked to obesity. However, long-term use of anti-obesity medications causes serious side effects, including regaining weight after stopping treatment (Christensen et al. 2007; Kang and Park 2012). Researchers are now focused on seeking alternative therapeutic strategies to overcome this economic and social problem and resolve the challenges of obesity. Due to a plethora of health-enhancing effects, natural dietary compounds such as mushroom polysaccharides play a significantly important role in the prevention of several ailments. The polysaccharides from mushrooms (prebiotics), which function as a substrate for gastrointestinal tract microorganisms, enhance microorganisms that have a positive physiological effect on the immunity of the host (Gibson et al. 2004; Rathore et al. 2017). Furthermore, dietary fiber promotes the growth of bacteria that produce short-chain fatty acids (SCFAs), which act as anti-inflammatory agents (Cani et al. 2007).

Traditional Chinese medicine (TCM) in Asia has a long history that dates back many years ago (Tang et al. 2008). For 2000 years on, in traditional Chinese medicine, Malaysia and Japan have eaten *Morchella* species mushrooms as a cure for various diseases (Hobbs 1995). *Morchella esculenta* is an edible mushroom known for its high nutritional value and delicious taste (Tietel and Masaphy 2018). It contains a wide range of bioactive substances that have traditionally been used in medicine (Ferreira et al. 2010). A plethora of studies has revealed *Morchella esculenta* numerous biological properties, including neuroprotective and nephroprotective (Tietel and Masaphy 2018). The anti-proliferating ability of *M. esculenta* polysaccharides against human colon cancer (HT-29 cells) has been identified from *M. esculenta* Exopolysaccharide, which possesses tumor-suppressive effects in vitro (Hu et al. 2013). In addition, *M. esculenta* demonstrated immunostimulatory function by stimulating T cells and proliferating splenocytes (Cui et al. 2011). The anti-obesity impact of MEP by regulating HFD-induced obesity and inflammation via enhancing the gut microbiome population is yet to be investigated. In the present research, considering the beneficial role of *Morchella esculenta* mushroom in preventing multiple health issues, we also studied the impact of MEP on obesity and inflammation induced by HFD.

## Materials and methods

### Chemicals and reagents

The fruiting body of *M. esculenta* mushroom was obtained from Shandong Tai’an Ynsheng Food Co., Ltd Shandong, China. The high-fat diet of 60% was purchased from Jiangsu Medison Biomedical Co., Ltd., (Yangzhou, China). The faecal DNA extraction kit (FORGENE) was from Chengdu, China, and Beckman Coulter (Brea, CA, USA). The bicinchoninic acid (BCA) protein assay kit and all enzyme-linked immunosorbent assay (ELISA) kits were purchased from TransGene Biotech (Beijing, China) and Shanghai Longton Biotechnology (Shanghai, China) respectively. All the primary and secondary antibodies goat anti-rabbit [β-actin, zonula occludens-1(ZO-1), claudin-1, zonula occludens-2 (ZO-2), occludin, mucin-2 (MUC2) p65 and p-p65], and the Radioimmunoprecipitation assay (RIPA) reagent were supplied by Proteintech, Wuhan, China, while tumor necrosis factor-alpha (TNF-α), interleukin-6 (IL-6), iNOS, COX2, TLR4, and IκκB were from CUSABIO, Wuhan Donghu Hi-Tech Development Area, Wuhan, Hubei Province, China. Immobilon-P Polyvinylidene difluoride (PVDF) membranes were from Life Science Research, USA, while the ECL (enhanced chemiluminescence) substrate was obtained from Advansta, Inc., Menlo Park, CA, USA. The PrimeScript RT-PCR Kit was from the USA.

### Mice and study design

**Fig. 1:**
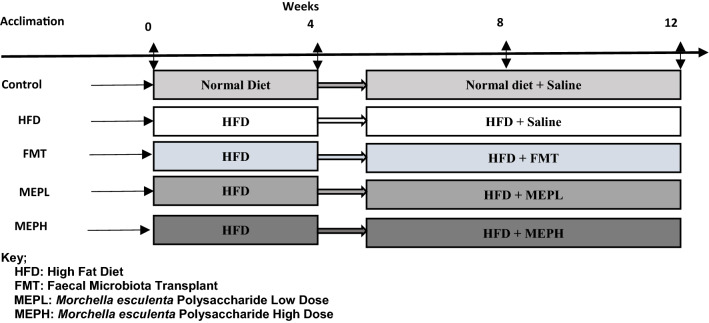
Experimental plan. Mice were divided into five groups (n = 8 in each group). Normal mice (control group), high fat diet (HFD group), fecal microbiota transplant (FMT group), low dose of MEP (MEPL group), and high dose of MEP (MEPH)

Forty male BALB/c mice weighing 15 ± 2 g were obtained from the animal care center specific-pathogen-free (SPF) with the approval of Dalian Medical University (Animal Care and Research Ethics Committee, Dalian, China) (Approval Number: ARYX 2019–2021). All mice were randomly assigned to groups and housed in environmentally friendly conditions (22 ± 2 °C temperature, 50 ± 10% relative humidity, and a 12 h light-dark cycle), with free access to normal diet and water. After 1 week of acclimation, mice were distributed into five groups (each group containing eight mice). One group was fed a normal chow diet, and the remaining four groups of mice were fed a 60% high-fat diet (HFD) for 4 weeks (Fig. 1). From the 5th week, the normal diet (control) and high-fat diet (HFD) groups were administered with saline (200 µl). *M. esculenta* polysaccharide (MEP) was gavaged (200 µl) daily at a low dose [MEPL group] of 200 mg/kg and a high dose [MEPH group] of 400 mg/kg of body weight orally till the 12th week. The treatment and dosage were performed according to the previous study protocol (Liang et al. 2018). For faecal microbiota transplant (FMT Group), the protocol was followed as established previously (Borody et al. 2013). Briefly, the stool of normal diet mice (control group) was pooled together and 100 mg resuspended in 1000 µl sterile saline. The mixed solution was centrifuged for 3 min at 800*g*. The supernatant was separated and gavaged via oral daily as faecal transplant materials of 100 µl (FMT group) till the 12th week. The schematic presentation and study design were shown in (Fig. 1). The body weights of all animals were weighed daily during MEP treatment. After 8 weeks of MEP treatment, the faecal samples were collected in Eppendorf (EP) tubes and stored immediately at − 80 °C. All the mice have been sacrificed. The blood samples, organs, and tissues were collected for further analysis.

### Extraction and characterization of mushroom polysaccharides (MEP) from the *M. esculenta* mushroom

The crude polysaccharide from *M. esculenta* mushroom fruiting bodies was extracted according to the protocol previously reported (Kanwal et al. 2018). The dried fruiting bodies of *M. esculenta* were crushed into a fine powder and mixed with distilled water at a ratio of 1:50 g/ml, then heated at 80 °C for 3 h. The mixture was deproteinized with trichloroacetic acid at 1.5% (v/v), pH was adjusted to 7.0 by 2 M (NaOH), and centrifuged for 10 min at “10,000*g*”, and the supernatant was collected and concentrated by rotary evaporation at 65 °C. The concentration of protein was measured by the BCA quantification method. The concentrated solution was precipitated by 3 volumes of ethanol at 4 °C for 12 h and then freeze-dried under freeze-drying vacuum systems. The carbohydrate content of *M. esculenta* crude polysaccharide (MEP) was determined through the phenol-H_2_SO_4_ method (Dubois et al. 1956). And for the determination of the monosaccharide composition, a high-performance liquid chromatography (HPLC) technique was used (Hao et al. 2016). In brief, 50 mg of purified polysaccharide powder was hydrolyzed at 120 °C for 6 h with 2 mol/L of trifluoroacetic aqueous solution. After hydrolysis, the excess acid was removed by co-distillation with methanol to yield a dry hydrolysate, which was dissolved in NaOH and methanol solutions and then incubated for 1 h at 70 °C. After pH normalization, distilled water was added and the mixture was extracted three times with chloroform and filtered through a 0.22 μm nylon membrane (Westborough, MA, USA).

### Morphometry assessment of adipose tissues

For adiposity assessment, freshly dissected adipose tissues (epididymal) from all groups of mice were fixed in 10% formalin for 12 h. The 4 mm sections were prepared by deparaffinized, rehydrating, and staining with hematoxylin and eosin. The morphological examination was performed under the microscope (Leica Microsystems, Wetzlar, Germany). The number of adipocytes from each sample of different study groups was counted, and the adipocyte size was evaluated via Image J (NIH, USA) as reported previously (Parlee et al. 2014).

### Biochemical analysis

The mice were humanly sacrificed by cervical dislocation after 8 weeks of experimental duration. By extirpating the eyeball, blood was collected, and serum was separated by centrifuging at 1000*g* for 10 min. The serum of all experimental groups was subjected to the HITACHI 7600 automatic biochemistry analyzer (Hitachi High-Technologies Co., Tokyo, Japan) to determine the concentration of aspartate aminotransferase (AST), alanine aminotransferase (ALT), triglycerides (TG), and total cholesterol (TC). A fasting blood glucose (FBG) level was measured by a glucometer via puncturing the tail vein of mice. The serum insulin levels and non-esterified fatty acids (NEFA) were measured by an ELISA kit according to the protocol of the manufacturer.

### Cytokine concentration measurement

The serum concentration of endotoxin lipopolysaccharide (LPS), anti-inflammatory cytokine interleukin 10 (IL-10), and pro-inflammatory cytokines [interleukin 1β (IL-1β), IL-6, and TNF-α] were determined by using the mouse ELISA kit (enzyme-linked immunosorbent assay) (Shanghai Longton Biotechnology Co., Ltd., Shanghai, China), following the manufacturer’s guidelines.

### RNA extraction and quantitative real-time reverse-transcription polymerase chain reaction (qRT-PCR)

The RNA from adipose tissue was isolated using the TRIzol reagent (Invitrogen Life Technology, Gaithersburg, MD, USA) by following the manufacturer’s guidelines. The Real-Time PCR thermocycler (Applied Biosystems StepOnePlusTM) was used while employing SYBR Green (Kapa SYBR Fast Master Mix) in triplicate to perform qRT-PCR. A reaction mixture of a total volume of 10 µl was used for each tube containing 1 µl of target primers, 1.5 µl of cDNA, 5 µl of SYBR Green Master Mix, and 2.5 µl of nuclease-free water. RT-PCR was used for 50 cycles, with the following PCR conditions: pre-incubation at 95 °C for 10 min; denaturation at 94 °C for 15 s; annealing at 60 °C for 30 s; and elongation at 72 °C for 30 s. The relative expression of the gene was quantified using method 2–DDCt (Livak and Schmittgen 2001) while using β-Actin as an internal control. The sequence details of the primers are shown in (Additional file 1: Table S1).

### Western blotting analysis

Total colonic protein was extracted by radioimmunoprecipitation assay using RIPA lysis buffer with protease inhibitor. After centrifugation of 12,000*g* at 4 °C for 5 min, the protein was quantified by the BCA protein assay kit. An equal amount of protein lysate was fractionated by 8–12% sodium dodecyl sulphate–polyacrylamide gel (SDS-PAGE) and electro-blotted onto PVDF (polyvinylidene difluoride) membranes. Membranes were blocked by non-fat milk (5%) buffer, TBS (Tris Buffered Saline) at room temperature for 1.5 h. The membranes were then incubated overnight at 4 °C with their respective primary antibodies (1:500–1000), with β-actin (1:5000) serving as an internal control. Membranes were washed with TBS followed by incubation with horseradish peroxidase-conjugated secondary antibodies (1:5000) for 1 h at room temperature. Finally, the protein bands were exposed to an enhanced chemiluminescent (ECL) substrate (Millipore) and visualized using the Automated Imaging System.

### Genomic DNA extraction, 16SrDNA amplicon pyrosequencing analysis

Bacterial genomic DNA samples were extracted from colon content using the Power Max (stool/soil) DNA isolation kit (MoBio Laboratories, Carlsbad, CA, USA), following the manufacturer’s instructions, and stored at 20 °C before further analysis. The extracted genomic DNA was quantified by NanoDropND-1000 (Thermo Fisher Scientific, Waltham, MA, USA) while the quality of the DNA was determined through agarose gel electrophoresis and spectrophotometer, respectively. The 16srRNA gene V4 region was amplified by PCR using the forward primer 515F (5′-GTGCCAGCMGCCGCGGTAA-3′) and the reverse primer 806R (5′-GGACTACHVGGGTWTCTAAT-3′) with the following protocol: Thermal cycling consisted of 30 s of initial denaturation at 98 °C, followed by 25 cycles of 15 s of denaturation at 98 °C, 15 s of annealing at 58 °C, and 15 s of extension at 72 °C, with a final extension of 1 min at 72 °C.PCR amplicons were purified with Agencourt AMPure XP beads (Beckman Coulter, Indianapolis, IN) and quantified using the Pico Green dsDNA Assay Kit (Invitrogen, Carlsbad, CA, USA). The amplicons were then pooled in a normalized manner and sequenced using the [“IlluminaNovoSeq6000” platform format by GUHE Info Technology Co., Ltd (Hangzhou, China)] with a pair-end 2 × 150 bp. The Illumina sequencing raw data was submitted to the National Center for Biotechnology Information (NCBI) as a BioProject with the accession number PRJNA809155. The Quantitative Insights of Microbial Ecology (QIIME, v1.9.0) pipeline was used to process the sequence data. By eliminating the low-quality reads, the correct sequences were identified and the Operational Taxonomic Unit (OTU) was picked, including dereplication, cluster, and chimera detection using V search V2.4.4. Subsequently, OTU taxonomy classification was performed by the Green Gene Database with their representative sequences. In addition, QIIME and R packages (v3.2.0) were used to perform OTU-level alpha diversity, Simpson, Shannon diversity, evenness, and richness indices, while beta diversity was performed via UniFrac distance metrics and visualized through principal coordinate analysis (PCoA), non-metric multidimensional scaling (NMDS), and principal component analysis (PCA) (Caporaso et al. 2010).

### Statistical analysis

Statistical analysis was performed by the software GraphPad Prism 8.01 (La Jolla, CA, USA). A one-way analysis of variance (ANOVA) was performed with Tukey’s multiple comparison post hoc test to determine the significance of differences, and a value of 0.05 was considered statistically significant.

## Results

### Characterization and chemical analysis of *M. esculenta* polysaccharide (MEP)

The polysaccharide of *M. esculenta* (MEP) was extracted and the concentration was found to be 11.96 mg/ml by using the phenol-sulphuric acid method. The percentage yield was 13.5%; the total polysaccharide and protein contents were 96% and 2.26%, respectively. The monosaccharide composition analysis of (MEP) is shown in (Table 1). The findings from HPLC regarding monosaccharide composition are shown in Additional file 1: Fig. S1.

**Table 1:** Monosaccharides composition of *M. esculenta* polysaccharide (MEP)

Components	Concentration, mg/kg	Percentage, %
Mannose	42334.69	5.77
Ribose	1924.99	0.263
Rhamnose	86.36	0.018
Glucuronic acid	261.74	0.036
Galacuronic acid	42.91	0.006
Glucose	596658.04	81.35
Galactose	25981.32	3.543
Arabia sugar	65966	8.99
Fucose	113.73	0.016

### MEP treatments ameliorated mice HFD-induced obesity

**Fig. 2:**
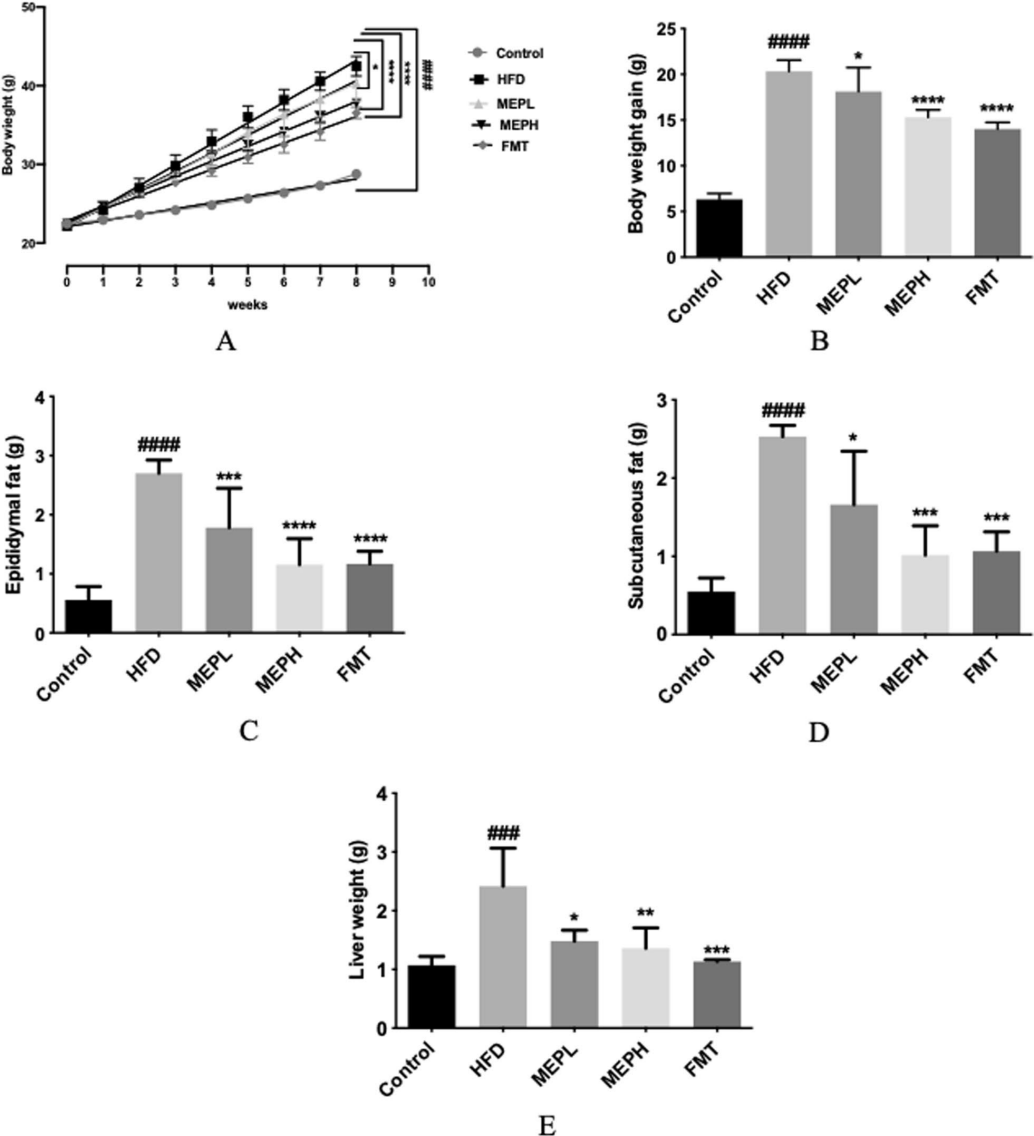
Effect of MEP on body weight and fat deposition in HFD-induced obesogenic mice. **A** body weight (g) during 8 weeks treatment with MEP, **B** body weight gain in (%), **C** epididymal fat (g), **D** subcutaneous fat (g), and **E** liver weight (g), data expressed as mean ± SEM (n = 8). By using one-way analysis of variance (ANOVA) with Tukey’s multiple comparison, significant differences were presented using ^#^p < 0.05, ^##^p < 0.01 and ^###^p < 0.001 ^*#*###^p < 0.0001 vs. Control group and *p < 0.05, **p < 0.01and ***p < 0.001 ****p < 0.0001 vs. HFD group

After MEP supplementation, the obesity parameters (body and liver weight, epididymal, and subcutaneous fat deposition) in the HFD group showed significant increments as compared to the control group (Fig. 2). Our results demonstrated a significant decrease in body and liver weight, as well as epididymal and subcutaneous fat deposition in the FMT and MEP treatment groups as compared to the HFD group (Fig. 2A and B). The HFD group body weight (42.53 ± 1.8 g) showed a significant increment as compared to the MEPH (37.78 ± 0.49 g), MEPL (36.6 ± 2.07 g), and (40.37 ± 0.70 g) groups, respectively (Fig. 2A). A significant reduction in weight gain has been observed upon 8 weeks of MEP supplementation in the MEPH group (p < 0.0001), the MEPL group (p < 0.05), and FMT (p < 0.0001) (Fig. 2B). While the MEP and FMT treated groups showed a significant reduction in subcutaneous and epididymal fat deposition (Fig. 2C and D). However, the weight of the liver was significantly increased in the HFD group (p < 0.001) as compared to the control group. However, the liver weight was significantly reduced in the MEPH group (p < 0.01), the FMT group (p < 0.001), and the MEPL group (p < 0.05) respectively (Fig. 2E). These results show that MEP controls weight gain and fat deposition in HFD mice.

### Effect of MEP on the histology of adipose tissue and expression of lipogenic and adipogenic genes

**Fig. 3:**
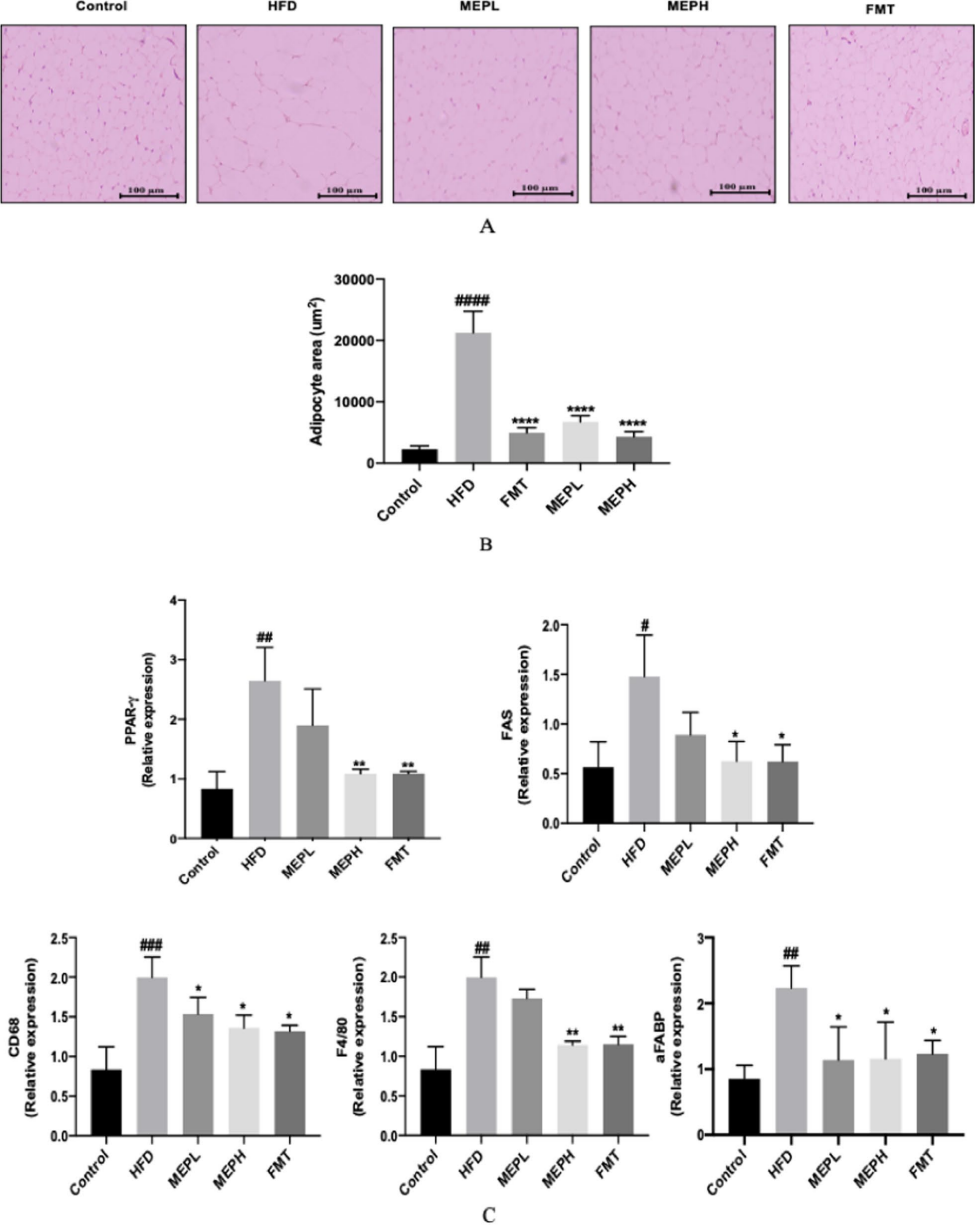
MEP effects on adipose tissue (epididymal) histology, lipogenic and adipogenic gene expression. Adipocyte morphology was analyzed by H&E staining and the size of the adipocyte was estimated via ImageJ software. 100 mm scale bar. **A** The variations of adipocyte histology in different treatment groups are shown in H & E images. **B** Adipocyte area distribution suggests that HFD has a larger area of adipocytes (> 20,000 mm^2^). In comparison, the MEP-supplemented groups showed smaller adipocytes (20,000 mm2) in size. **C** qRT-PCR was used to determine the effect of MEP treatment on the mRNA expression levels of lipogenic and adipogenic genes PPARy, aFABP, FAS, CD68, and F4/80 in adipose tissue. Data are presented as mean ± SEM (n = 8). By using One-way analysis of variance (ANOVA) with Tukey’s multiple comparison, Significant differences were presented using at ^#^p < 0.05, ^##^p < 0.01, ^###^p < 0.001, ^*#*###^p < 0.0001 vs. Control group and *p < 0.05, **p < 0.01, *** p < 0.001 **** p < 0.0001 vs. HFD group

Upon administration of MEP, body weight, epididymal, and subcutaneous fat deposition of HFD mice were significantly reduced. We further examined the adipocyte size in the HFD-fed, MEP-treated, and FMT-treated groups. The histological examination showed the level of lipid deposition in the adipose tissue, which would have been proportional to the size of the tissue. The adipocyte size in the HFD group (p < 0.0001) was significantly larger as compared to the control group. A significant reduction in adipocyte size occurred in the MEPH, MEPL, and FMT groups (p < 0.0001) (Fig. 3A and B). Our finding suggests the regulatory effect of MEP on fat deposition, which is in agreement with the previous finding that fat deposition was studied in HFD-fed mice (Chang et al. 2018). The overexpression of adipogenic genes (PPAR "g", aFABP, and FAS) was significantly increased in the HFD group (p < 0.01, p < 0.00, p < 0.001, p < 0.05) as compared to the control group (Fig. 3C). The levels of mRNA expression of CD68 (p < 0.01) and F4/80 (p < 0.001) markers of macrophages were also significantly increased in HFD mice as compared with the control group (Fig. 3C). However, MEP and FMT group treatments, specifically in the MEPH group, significantly decreased the expression level of adipogenic genes.

### Effect of MEP on glucose, insulin levels, lipid profile, and liver markers in obese mice

**Fig. 4:**
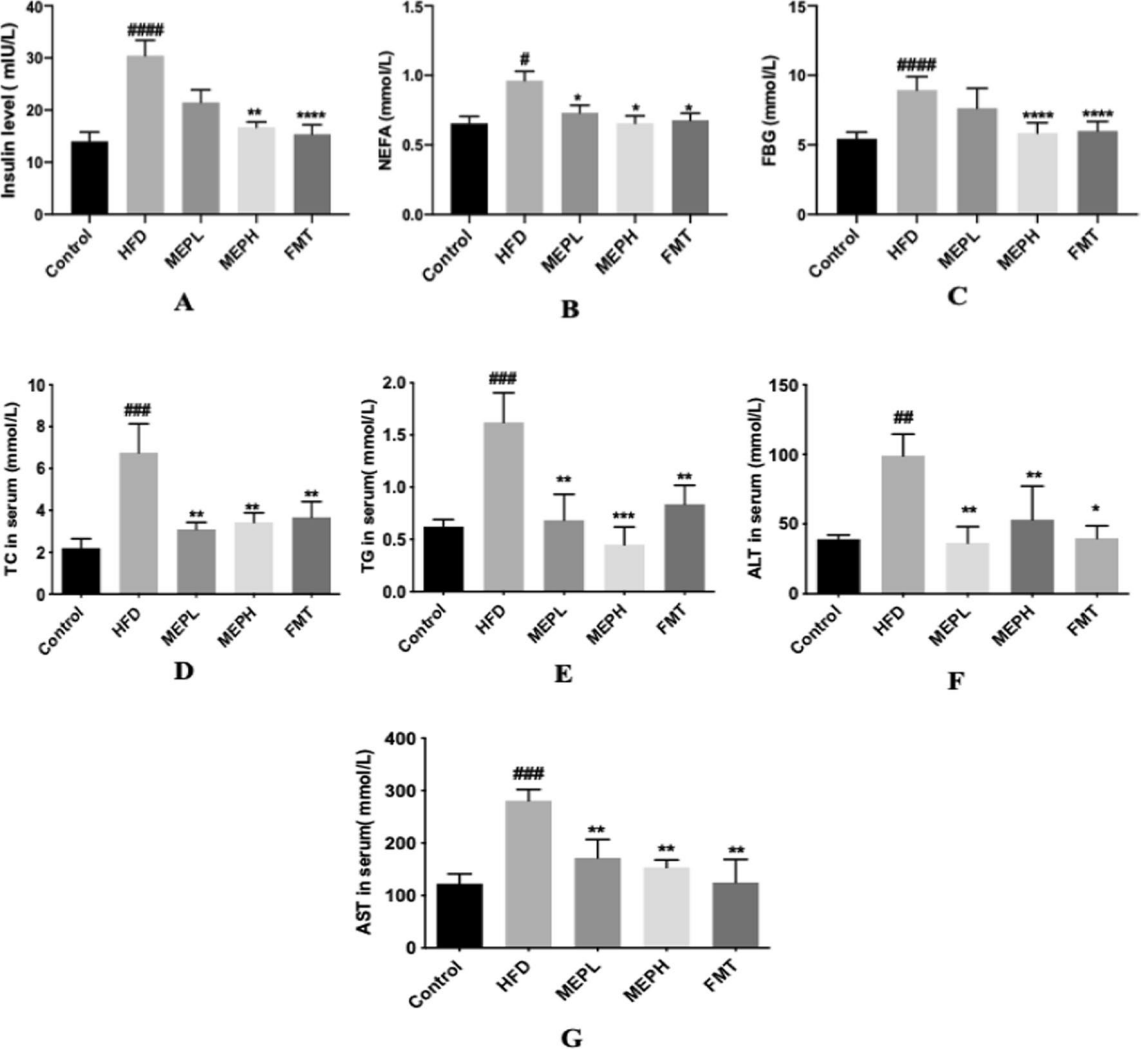
MEP effect on serum levels of insulin, NEFA, FBG, TC, TG, ALT, and AST in HFD-induced obese mice. **A** Insulin, **B** FBG, **C** NEFA, **D** TC, **E** TG, **F** ALT and **G** AST. Data are presented as mean ± SEM (n = 8). By using one-way analysis of variance (ANOVA) with Tukey’s multiple comparison, significant differences were presented using ^#^p < 0.05, ^##^p < 0.01 and ###p < 0.001, ^####^p < 0.0001 vs. Control group and *p < 0.05, **p < 0.01 and ***p < 0.001 ****p < 0.0001 vs. HFD group

We studied the effects of MEP treatment on glucose, insulin, TC, TG, ALT, AST, and NEFA (Fig. 4A–F). To investigate the MEP effect on the homeostasis of glucose, we determined the levels of fasting blood glucose and fasting insulin. Our results revealed that the HFD group showed a significant increment in fasting blood glucose and insulin levels as compared to the control group, However, MEP treatment significantly controlled the level of fasting glucose and fasting insulin (Fig. 4A and B). Our findings further demonstrated that the HFD group exhibited a significant increment of serum TC, TG, AST, ALT, and NEFA (p < 0.01) as compared with the control group (Fig. 4C–F). However, MEP treatment reduced the level of TC in MEPH (p < 0.01), MEPL, (p < 0.01) and FMT (p < 0.01), TG level in MEPH (p < 0.001), MEPL (p < 0.01), and FMT (p < 0.01). Moreover, liver markers such as ALT in MEPH (p < 0.01), MEPL (p < 0.01) and FMT (p < 0.05), and TG in MEPH (p < 0.01), MEPL (p < 0.01), and FMT (p < 0.01). NEFA levels in MEPH (p < 0.05) and FMT (p < 0.05) were also significantly reduced.

### Effect of MEP on endotoxemia and tight junction-related proteins (TJPs) in obesogenic mice

**Fig. 5:**
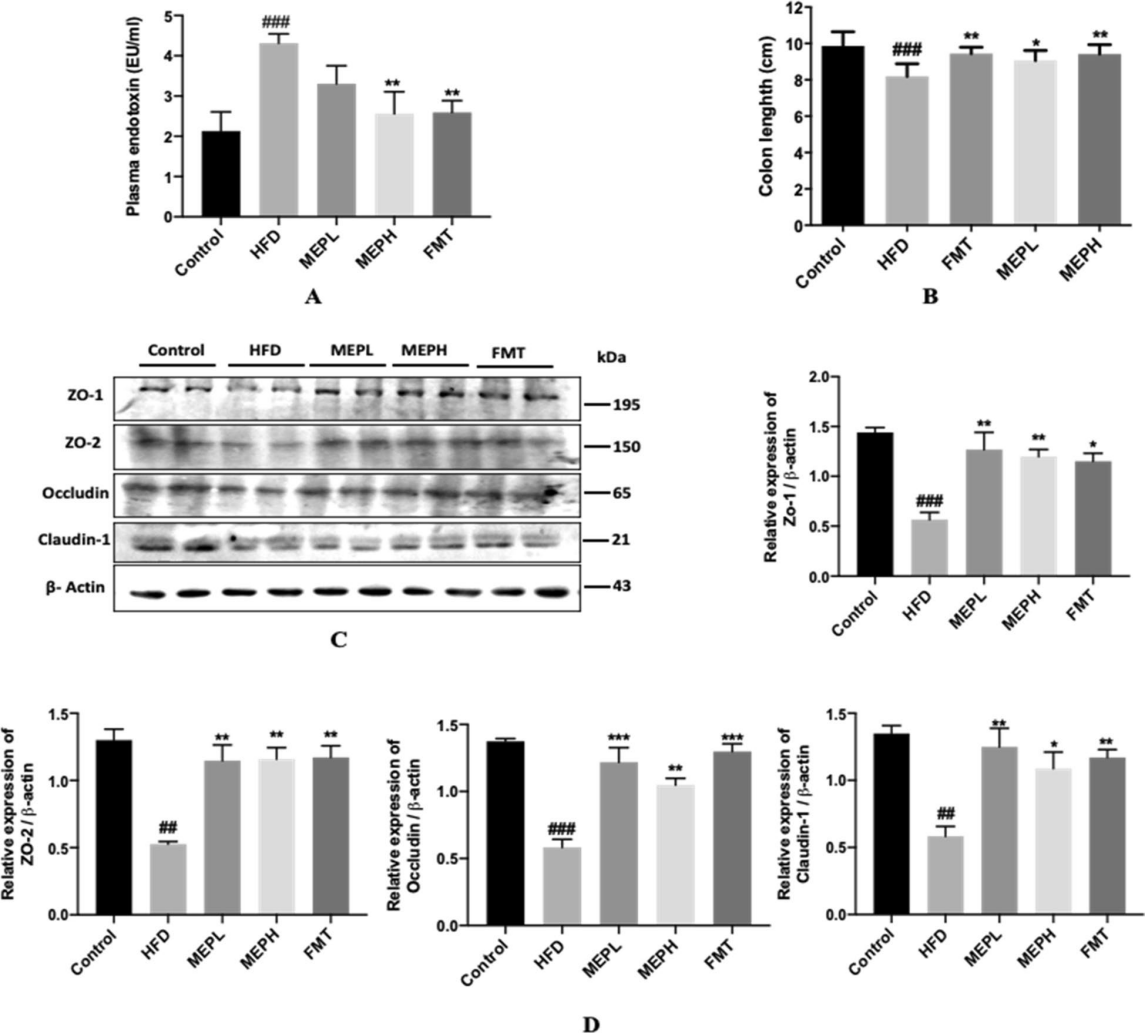
Effect of MEP on endotoxemia and Tight Junction-related Protein (TJP) in obesogenic mice. **A** The colon’s length was measured in centimeters. **B** The effect of MEP treatment on LPS levels of serum endotoxin (EU/ml) was measured by the limulus amebocyte lysate test kit. **C** TJP (ZO-1, ZO-2, occludin, and claudin-1) immunoblots from colon tissues in different MEP treatment groups. **D** Bar graph shows the relative band strength of the respective protein against β-actin as an internal control quantified by image J software. The data was obtained by three independent experiments and is expressed as mean ± SEM (n = 8). Significant differences were presented using at ^#^p < 0.05, ^##^p < 0.01, ^###^p < 0.001 and ^####^p < 0.0001 vs. Control group and *p < 0.05, **p < 0.01 and ***p < 0.001 and **** p < 0.0001 vs. HFD group

The impact of the MEP on the level of serum endotoxin was measured. Our results demonstrated that serum endotoxin levels in the HFD group (p < 0.001) elevated significantly compared to the control group (Fig. 5A). While the MEPH group (p < 0.01) and FMT group (p < 0.01) treatments led to significantly reduced LPS levels, we analyzed the impact of MEP on obesity-associated intestinal inflammation induced by HFD. The macroscopically examined colon did not have any significant changes among the groups. However, HFD was associated with intestinal inflammation, revealed by shrinkage of colon size. The HFD group observed a significantly shortened (p < 0.001) colon size compared with the control group (Fig. 5B). Nonetheless, the MEPH group (p < 0.01), MEPL group (p < 0.05), and FMT group (p < 0.01) observed a significant increment in colon size.

We further analyzed the efficacy of MEP on tight junction-related proteins (TJP). The HFD group showed reduced TJP expression levels compared to the control group (Fig. 5C and D). However, MEP treatment showed an increment in the expression levels of TJP between all groups (MEPH, MEPL, and FMT). Our results revealed that MEP treatment modulated intestinal permeability.

### Modulatory effects of MEP on inflammatory-related (TLR4) toll-like receptor 4 signaling pathway

**Fig. 6:**
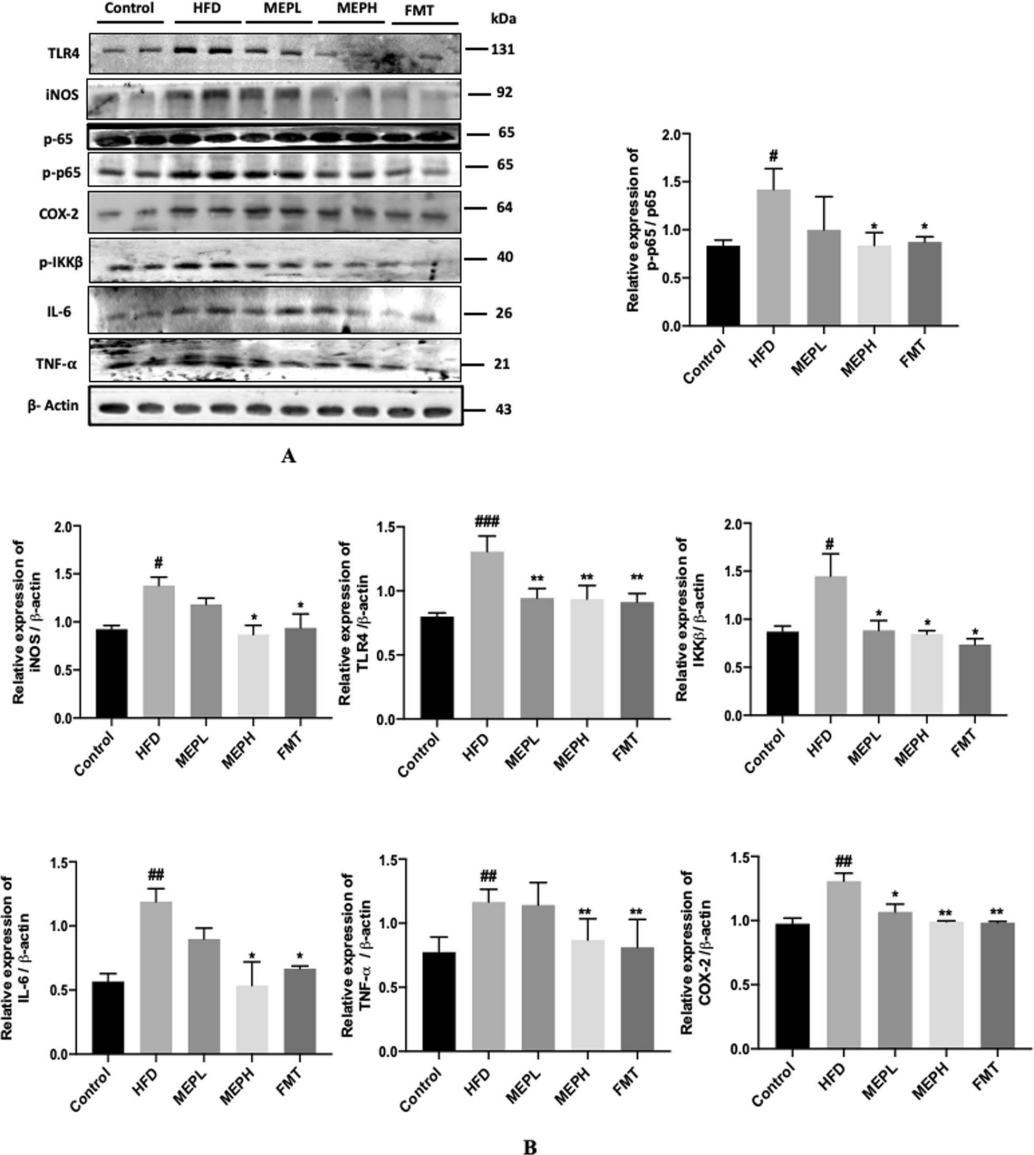
Effect of MEP on the regulation of inflammatory-related signalling pathways. **A** Western blot analysis of TLR4, iNOS, COX-2, p-IKKβ, p-p65, TNF-α, and IL-6 protein expression levels in the colon. **B** Bar graph showing quantification of the western blot of the respective protein against β-actin as an internal control by image J software. The data was obtained by three independent experiments and is expressed as mean ± SEM (n = 4). Significant differences were presented using ^#^p < 0.05, ^##^p < 0.01, ^###^p < 0.001 and ^####^p < 0.0001 vs. Control group and *p < 0.05, **p < 0.01, ***p < 0.001 and ****p < 0.0001 vs. HFD group

The effects of MEP on inflammatory-related TLR4 signaling pathways in intestinal tissue were determined by immunoblotting. Our results demonstrated that the TLR4 expression of the HFD group (p < 0.001) significantly increased compared with the control group (Fig. 6A and B). However, MEP treatment significantly decreased TLR4 expression in the MEPH group (p < 0.01), MEPL group (p < 0.01), and the FMT group (p < 0.01) as compared to the HFD group. The NF-κB p65 phosphorylation of the HFD group was also increased significantly (p < 0.05) as compared with the control group, showing the activation of NF-κB (Fig. 6A and B). The p65 phosphorylation was significantly decreased in the MEPH group (p < 0.05) and the FMT group (p < 0.05) upon MEP treatment. Inflammatory markers like iNOS, COX-2, IL-6, and TNF-α have previously been shown to cause intestinal damage by promoting inflammatory responses. When compared to the control group, the HFD group showed an increase in iNOS, COX-2, IL-6, and TNF-α expression (p 0.05) (Fig. 6A, B). MEP administration significantly reduced iNOS, COX-2, TNF-α, and IL-6 overexpression in the MEPH (p 0.05) and FMT (p 0.01) groups when compared to the HFD group. It has been demonstrated that MEP may modulate HFD-induced inflammation in mice via modulating TLR4 inflammatory-related signalling pathways.

### Effect of MEP supplementation on inflammatory circulatory cytokines in HFD-induced obese mice

**Fig. 7:**
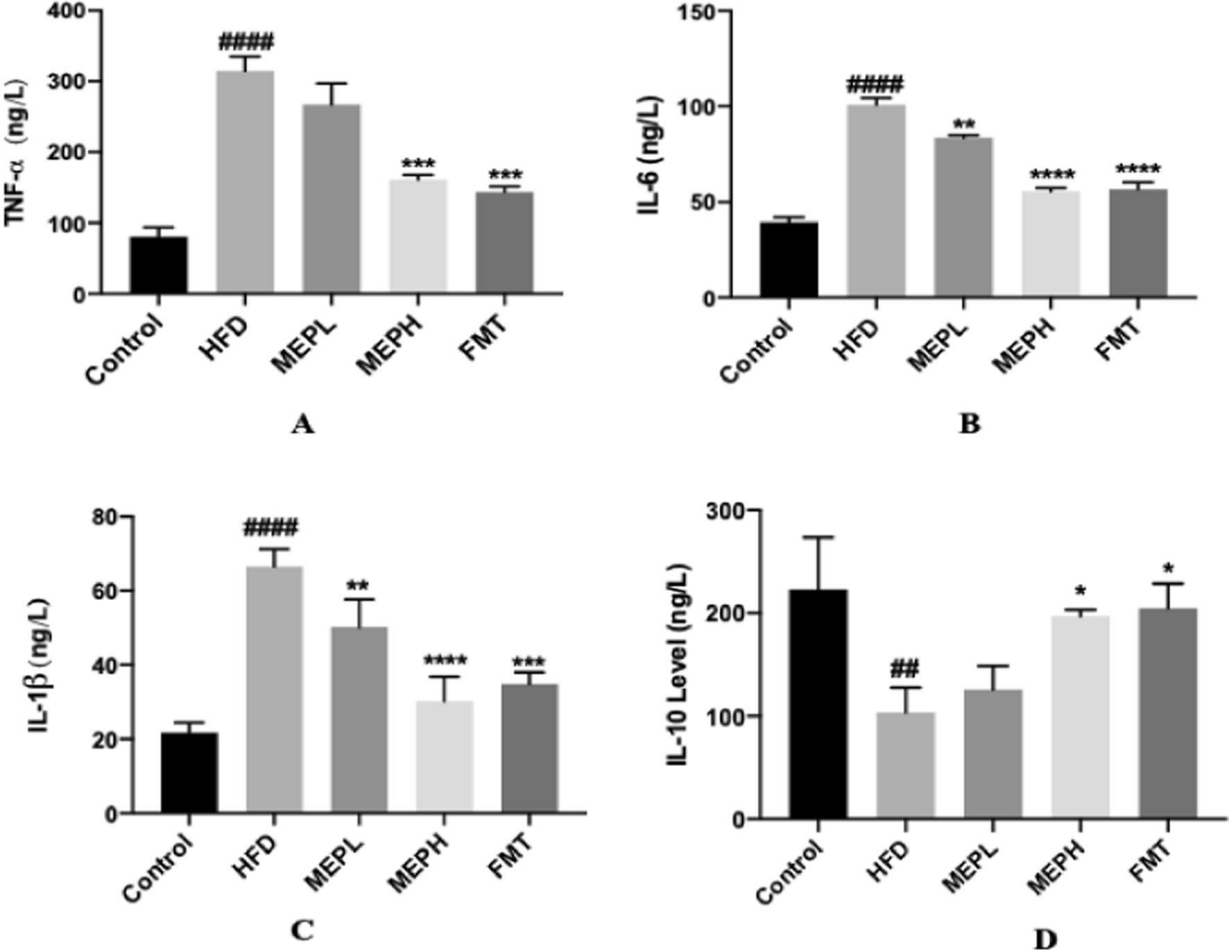
Effect of MEP on inflammatory cytokines in HFD-induced obese mice. **A–C** Pro-inflammatory cytokine levels of TNF-α, IL-6 and IL-1β. **D** The levels of anti-inflammatory cytokines and IL-10 levels in serum were determined by ELISA kits. The data was obtained by three independent experiments and is expressed as mean ± SEM (n = 4). Significant differences were shown using at ^#^p < 0.05, ^##^p < 0.01, ^###^p < 0.001 and ^####^p < 0.0001 vs. Control group and *p < 0.05, **p < 0.01, ***p < 0.001 and ****p < 0.0001 vs. HFD group

The effects of MEP on pro-inflammatory cytokines including TNF-α, IL-1β, and IL-6 as well as anti-inflammatory cytokines such as IL-10 were determined in different treated groups. We have found a significantly higher concentration of pro-inflammatory cytokines (IL-1β, TNF-α, and IL-6) in the HFD group versus normal mice (Fig. 7A–C). The anti-inflammatory circulatory cytokine (IL-10) concentration was significantly lower in the HFD group (Fig. 7D). Nonetheless, MEP supplementation significantly decreased the concentration of pro-inflammatory circulatory cytokines and increased anti-inflammatory circulatory cytokines in MEP and FMT-treated groups. These results indicate that MEP treatment reduces inflammatory markers in HFD-induced obesogenic mice.

### MEP supplementation effect on gut microbiota in HFD-induced obese mice

**Fig. 8:**
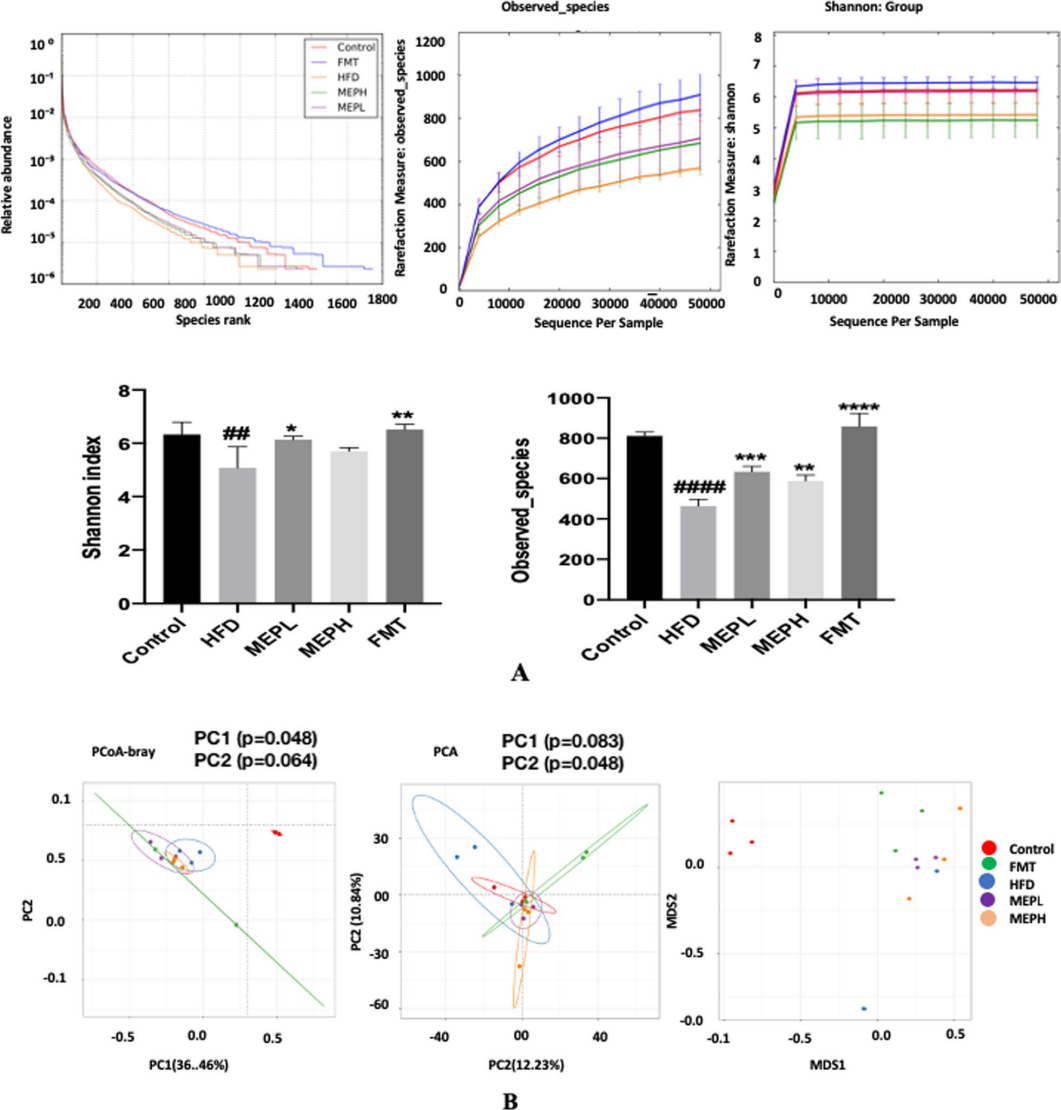
Effect of MEP on alpha and beta diversity indices in HFD-induced obese mice. **A** Alpha diversity was shown in the Rank abundance curve, observed species, and Shannon. The rank abundance curve indicates microbial richness and abundance, respectively. **B** The PCoA, PCA, and NMDS plots were used to assess beta diversity. Every point represents each sample independently. Points with various colours suggest different treatment groups. The distance between the various points represents the similarities and variations of the microbial population structure

Stool specimens were collected from HFD-induced mice after 8 weeks of MEP supplementation to study the overall changes in the composition of the gut microbiota using pyrosequencing (16 S rRNA gene). The alpha and beta diversity of each group was evaluated to determine the bacterial richness, abundance, diversity, and structural changes. As revealed via rarefaction curves (Fig. 8) and the number of sequences analysed, estimated OTU richness and coverage (Additional file 1: Table S2), and observed species (Fig. 8A), the bacterial richness and diversity of the HFD group was found to be different as compared to the control, MEPH, MEPL, and FMT groups. The HFD group (p < 0.0001) observed a significantly lower Shannon index than the control group. While MEPL (p < 0.0001) and FMT (p < 0.0001) observed significantly higher Shannon indexes as compared to the HFD group (Fig. 8A), At the level of observed species, the HFD group (p < 0.0001) showed the lowest species as compared to the control group. Meanwhile, the observed species in the MEP-treated groups showed significantly higher species as MEPH (p < 0.0001), MEPL (p < 0.0001), and FMT (p < 0.0001) than in the HFD group (Fig. 8A). These changes between all groups were also confirmed by Boxplot, as shown in (Additional file 1: Fig. S3). The beta diversity was analyzed via non-metric multidimensional scaling (NMDS) plot analysis, principal coordinate analysis (PCoA), and principal component analysis (PCA), which revealed bacterial community structural variation among all groups. Significant differences among the groups were observed for PCA in PC1 (p = 0.083), PC2 (p = 0.048), and for PCoA in PC1 (p = 0.048) and PC2 (p = 0.064) (Fig. 8B). Our results demonstrated, that HFD group samples were clustered distantly from the control group. However, MEPH, MEPL, FMT groups, and the control group are relatively similar to the HFD group.

### MEP administration alters bacterial taxonomy in HFD-treated mice

**Fig. 9:**
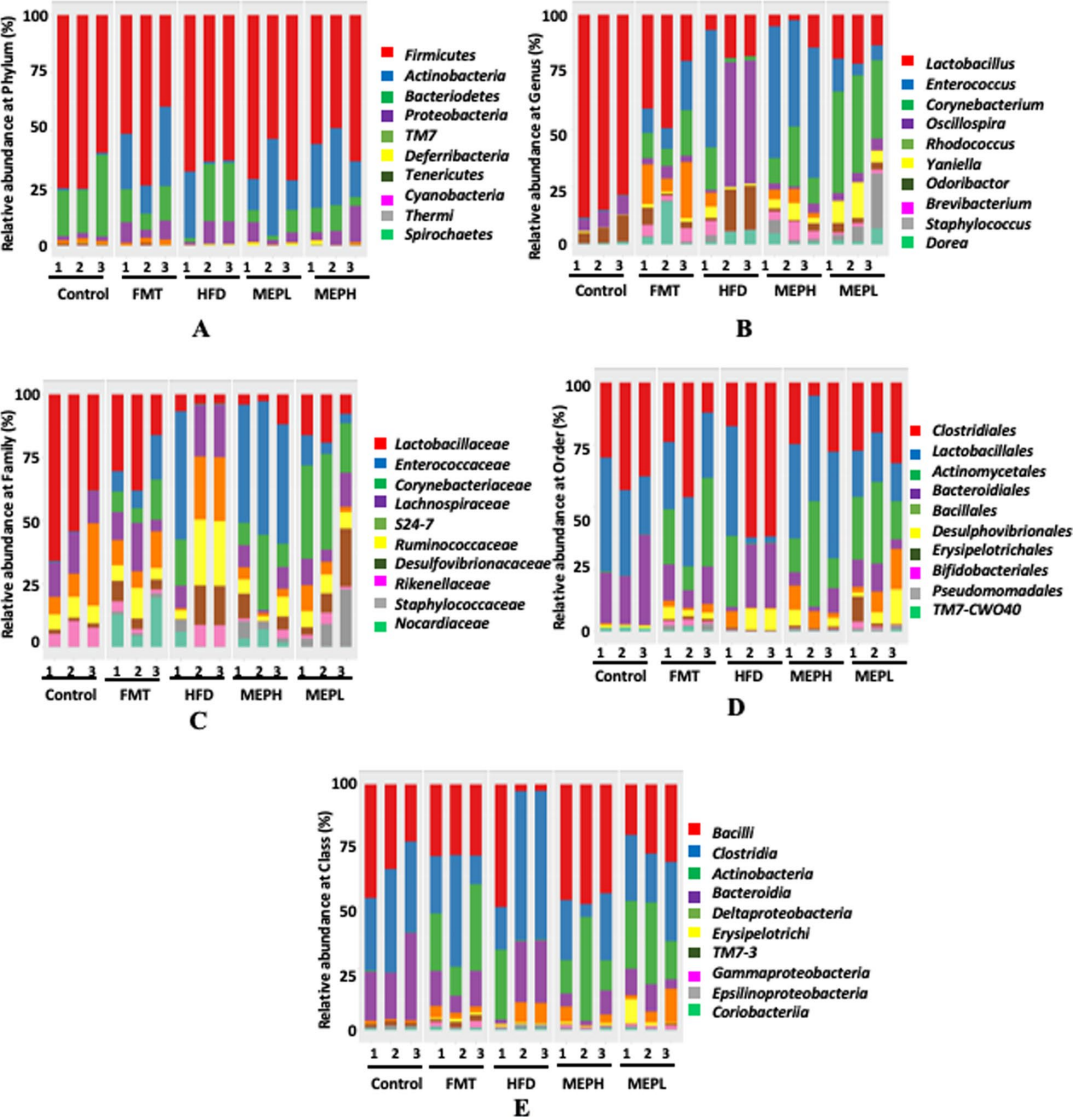
Bacterial composition at different taxonomy levels in HFD-induced obese mice. Relative abundance per cent (%) at **A** Phylum level, **B** Genus level, **C** Family level, **D** Order level, and **E** Class level, *Enterococcus, Lactobacillus, Corynebacterium, Rhodococcus* and *Oscillospira* were found in various treatment groups

At the phylum level, the alteration occurred in four main phyla: *Firmicutes, Bacteroides*, *Proteobacteria*, and *Actinobacteria* in the HFD group as compared to the control group (Fig. 9A). *Firmicutes* had a lower ratio (HFD group, 70.16% vs. control group, 62.37%), while *Bacteroidetes* had a higher ratio (HFD group, 17.09% vs. control group, 24.84%). However, the ratio of *Proteobacteria* showed an increment (HFD group, 10.02%) vs. the CON group, 2.01%) and *Actinobacteria* (HFD group, 10.50% vs. the CON group, 0.79%). MEP supplementation effectively attenuates the disturbing pattern in the MEP and FMT groups as compared to the HFD group shown in (Additional file 1: Table S3). Furthermore, the composition of bacterial flora in HFD mice changes at the class, family, order, and genus levels when compared to the control group (Fig. 9B–E). At the family level, the most abundant flora was *Lactobacilleace, Enterococcaceae, and Corynebacteriaceae, Lachnospiraceae, S24-7, Ruminococcaceae*, and *Desulfovibrionaceae.* Nonetheless, upon MEP supplementation, the altered bacterial flora has been reversed at various ratios. *Lactobacilleace* declined in the HFD alone group compared to the control group (HFD group, 53.13% vs. Control group, 4.18%) and *Enterococcaceae* (HFD group, 25.90% vs. Control group, 0.35%), which recovered upon MEP treatment as presented in (Additional file 1: Table S4).

At the genus level, *Lactobacillus* and *Enterococcus* were altered (HFD group, 92.56% vs. Control group, 7.78%) and *Enterococcaceae* (HFD group, 0.25% vs. Control group, 48.18%), respectively. These changes were ameliorated in the MEP and FMT treated groups (Fig. 9B and Supplementary Table. S4). At order and class level, *clostridiales* and *clostridia* drastically increased in the HFD alone group compared to the control, MEP, and FMT treated groups (Fig. 9D and E). The heat map of microbiome composition with clustering analysis of taxonomic level was clustered following the degree of similarity among all groups (Additional file 1: Fig. S4). Overall, the relative abundance of disrupted bacterial populations indicates improvement after MEP supplementation. Conversely, our findings also showed that at the taxonomic level, the bacterial population was perturbed in HFD mice. However, MEP and FMT administration in all groups enhanced the gut microbiota of HFD mice, and most of the bacterial flora was ameliorated to normal.

## Discussion

The potential role of medicinal and edible mushroom polysaccharides in the treatment of various diseases is well known (Chang et al. 2018). Among those, *M. esculenta* is an edible mushroom with many biological properties (Liu et al. 2016; Tietel and Masaphy 2018). The potential of the mushroom *M. esculenta* polysaccharides on obesity, inflammation, and gut microbiota has not been studied. Our study demonstrates that *M. esculenta* polysaccharides attenuate HFD-induced obesity and inflammation via ameliorating gut microbiota composition, inflammatory factors, and inflammatory-associated signaling cascades. Previous studies have shown that obesity has been mainly characterized by chronic inflammation and is often linked with the etiology of different chronic diseases, such as heart diseases, diabetes, inflammatory liver diseases, metabolic diseases, and tumors (Chang et al. 2015; Yoshimoto et al. 2013). Excessive fat deposition is known to be the key factor contributing to the progression of obesity (van Herpen and Schrauwen-Hinderling 2008). In our work, HFD consumption resulted in a significant increase in liver weight, body weight gain, and subcutaneous and epididymal fat. Moreover, the body and liver weight, epididymal, and subcutaneous fat deposition in the HFD group show significant increments as compared to the control group. On the other hand, MEP administration significantly reduces liver and bodyweight, as well as subcutaneous and epididymal fat deposition, when compared to the HFD alone group. These results demonstrate that MEP produces anti-obesity effects against HFD-induced obesity in mice. Previous research findings indicate that obesity caused by HFD is also characterized by hyperglycemia and hyperinsulinemia (Lu et al. 2019). In addition, we also found an elevated level of fasting insulin and glucose levels in our study. Interestingly, MEP treatment attenuated the increased levels of insulin and glucose at different doses in HFD-induced obese mice.

In several studies, the increase in the size of epididymal adipose tissue has been reported, and the number of adipocytes decreased in obese people and in-vivo (Chang et al. 2015). Furthermore, changes in transcriptional factor cascades, such as increased expression of adipogenic genes (PPAR-γ, aFABP, lipogenic genes (FAS), and macrophage-specific markers (CD68, F4/80), have been reported in obesity-related studies (Chang et al. 2015; Kim et al. 2012). Consistent with these findings, our results also show a larger size of adipocytes and over-expression of lipogenic, adipogenic genes, and macrophage-specific markers in the HFD alone group compared with the control group. While MEP treatment resulted in decreasing adipocyte size and also reducing the expression levels of lipogenic, adipogenic, and macrophage-specific markers.

Moreover, the development of obesity is attributed to the rise in adipocyte size owing to the secretion of pro-inflammatory cytokines (Kim et al. 2018). Previous research has shown that excessive production of pro-inflammatory cytokines (TNF-α, IL-1β, IL-6) causes chronic inflammation and insulin resistance (Qatanani and Lazar 2007). Therefore, it is necessary to regulate inflammatory cytokine secretion to prevent chronic inflammation and insulin resistance. In our study, we also observed the overproduction of pro-inflammatory cytokines (TNF-α, IL-1β, IL-6,) and the under-production of anti-inflammatory cytokines (IL-10) in HFD-induced obese mice. Notably, MEP treatment attenuated the inflammatory markers of HFD-induced obesogenic mice, which indicates their possible effects on inflammation. HFD consumption increases adiposity, which causes liver damage by increasing liver biomarkers (AST and ALT) as well as TG (Cui et al. 2011). NEFA comes from adipocytes that are related to metabolic disorders such as obesity. Adipocytes generate NEFA that affects the action of insulin and is also associated with metabolic disorders such as obesity (Delarue and Magnan 2007). In line with these results, we have also observed increased levels of (AST, ALT, TG, and NEFA) in HFD-induced obese mice. However, we found that MEP decreased these markers, indicating the effectiveness of MEP in the improvement of liver function and adiposity.

The TJ proteins play a role in epithelial permeability, cell-cell adhesion, and paracellular diffusion. In addition, TJ proteins such as cytoplasmic scaffolding proteins (ZO family) and transmembrane epithelial barrier proteins (Occludin and Claudins) play an important role in maintaining the integrity of the colon (Dokladny et al. 2016; Sánchez de Medina et al. 2014). Different inflammatory reactions affect the expression of TJ proteins. An increased concentration of pro-inflammatory markers (IL-1β and TNF-α) decreased the expression of TJs proteins in the colon (Al-Sadi et al. 2009). Considering that gut dysbiosis in HFD-induced obese mice may influence the efficiency of the intestinal permeability eventually, predisposing to the release of LPS into the bloodstream (Cani et al. 2008). Thus, HFD feeding contributes to intestinal inflammation and can cause obesity through increased plasma LPS levels, indicating a causative factor for intestinal inflammation in the emergence of obesity. Since TLR4 mediates LPS pro-inflammatory effects, there appears to be a correlation between the bacteria-related pro-inflammatory stats in the gut and the obesity development induced by diet (Ding and Lund 2011). Giving importance to the fact that TJs play a crucial role in preserving intestinal integrity, we have determined the expression of tight junction TJs proteins in the colon of HFD-induced obese mice. Consistent with this literature, our findings demonstrated the lower levels of TJs proteins in the colon of HFD-induced obese mice. However, MEP increased the expression levels of TJs in MEP-treated groups. These results have shown that MEP can improve histological changes and colon integrity by enhancing TJ proteins and mucin expression. In addition, we have also observed increased levels of LPS in HFD-induced obese mice. This may indicate a potential causative factor for gut inflammation in contributing to metabolic endotoxemia by perturbing gut permeability. While MEP treatment decreased LPS levels, which revealed that MEP could reduce endotoxemia in HFD-induced obese mice.

Previously, it was observed that higher concentrations of plasma LPS and increased endotoxemia induced by HFD were linked with increased expression of Toll-like receptor (TLR) 4 and NF-κB in mononuclear cells (Ghanim et al. 2009). NF-κB activation alleviates inflammatory reactions by up-regulating macrophage infiltration (Schreiber et al. 1998). Furthermore, it increases the expression of inflammatory enzymes such as iNOS and COX-2, as well as the expression of many cytokines such as (Chen et al. 2004; He et al. 2016). Adipose tissue produces increased levels of inflammatory markers, including TNF-α and IL-6 in dietary manipulated obesity models. Currently, macrophages of adipose tissue have been focused on, which are involved in adipose tissue by chemo-attractants and work as mediators to produce inflammatory reactions to obesity (Maury and Brichard 2010). Macrophages invade the adipose tissue, which leads to the expression of TNF-α, IL-6, and iNOS (Cancello et al. 2006). Our study confirms this series of pathways, as we observe increased expression of inflammatory cytokines and TLR4, iNOS, and COX-2. So this study is consistent with the previous finding that HFD induced inflammation through induction of TLR4 and activation of NF-κB. Our finding demonstrated the anti-inflammatory activity of MEP by NF-κB dephosphorylation, TLR4, iNOS, COX-2 inhibition IL-6, and TNF-α reduction respectively in MEP-treated HFD-induced obese group.

The gut microbiota has been the focus recently as several studies revealed the association between obesity-related and inflammatory disorders (Cani and Delzenne 2007; Tilg and Kaser 2011). The most important factors in obesity are the composition of the gut microbiota as it plays a key role in the acquisition of nutrients, the production of vitamins, and the regulation of fat and energy storage (Dethlefsen et al. 2007; Zhang et al. 2010). As a result, it investigated the relationship between obesity and gut microbiota to address obesity-related health issues. Diet has a major influence on gut microbiome composition. HFD feeding has been demonstrated to induce dysbiosis of the gut microbiota in obese people, and the disturbance of homeostasis of the gut microbiome has been related to the development of obesity and associated metabolic disorders (Ridaura et al. 2013). On the other hand, various studies have revealed a modulatory effect on the gut microbiome population of high fiber diets, including prebiotics, oligosaccharides, polysaccharides, and polyphenols (Chang et al. 2018; Etxeberria et al. 2013; Neyrinck et al. 2011). The modulation of microbial flora by fiber diets (prebiotics) and undigestible polysaccharides or oligosaccharides may have a positive effect on the microbial population by promoting the growth of useful microbial flora (Wilson and Whelan 2017).

Our findings demonstrate that polysaccharide treatment has positively improved the composition of the intestinal microbiota in MEP-treated groups. Previous research has demonstrated a decrease in bacterial richness and diversity in obese people relative to lean people (Le Chatelier et al. 2013; Ley et al. 2005). Consistent with the previous finding, our investigation yielded comparable results, namely; the HFD group had lower microbial diversity than the FMT and MEP-treated groups.

Furthermore, we investigated the gut microbiota composition at the taxonomy level. Previously, it was demonstrated in several studies that the gut microbiota of obese individuals and animals modals observed a disturbance in the *Firmicutes* to *Bacteroides* ratio, showing these phyla play a significant role in obesity-associated inflammation (Neyrinck et al. 2011). Other studies have found an increase in the *Firmicute*s to *Bacteroidetes* ratio in obese mice (Daniel et al. 2014; Ley et al. 2006). In our work, at the phylum level, the most abundant phyla were *Firmicutes, Proteobacteria, Bacteroidetes, Actinobacteria, TM7, Tenericutes*, and *Deferribacteres.* In consistent with the previous finding, the HFD group showed increased *Firmicutes* abundance and decreased *Bacteroides* abundance. However, MEP supplementation potentially ameliorates bacterial diversity at the phylum level, and we observed a relatively similar taxonomy between control, MEP, and FMT groups. We further analyzed the microbial population at the genus level. *Lactobacillus* was observed in a higher proportion in the control, MEP, and FMT groups, while *Enterococcus* proportion was increased in the HFD group. which showed that MEP positively enhanced the growth of *Lactobacillus* in HFD-induced obese mice. These results were in agreement with previous findings as the hypolipidemic effect was due to *lactobacillus* enhancement (Bhathena et al. 2009). Several species of *Lactobacillus* are known for the production of lactate, which is a precursor to short-chain fatty acids (SCFAs) (Tsukahara et al. 2002). *Enterococcus* expansion has also been linked with liver inflammation and inflammatory diseases such as inflammatory bowel diseases (IBD) (Kim et al. 2017; Um et al. 2013).

In order to avoid this social and economic catastrophe and solve issues related to obesity, scientists are now concentrating on developing novel treatment arsenals. Because of their numerous health-improving benefits, natural food components such as mushroom polysaccharides are playing a key role in the treatment of different illnesses. However, more study is required to purify and identify the bioactive constituents of crude polysaccharide from *M. esculenta* that are responsible for alleviating obesity and related parameters in a mouse model, as well as to assess the impact of MEP in clinical trials.

Altogether, MEP administration resulted in the attenuation of the obesity, inflammation, restores gut microbiota dysbiosis by enhancing beneficial bacteria like *Lactobacillus* and inhibiting pathogenic bacteria like *Enterococcus*. Furthermore, MEP supplementation reduced inflammation via Toll-like receptor 4 (TLR4) expression, improved gut integrity and reduced endotoxin levels in HFD-induced obese mice. We expect that the prebiotic effects of such bioactive polysaccharides could be made feasible in the immediate future.

## Supplementary Information

**Additional file 1.** Table S1: qRTPCR primers sequence; Table S2: Alpha diversity summary; Table S3: Bacterial phylum percentage in all groups; Table S4: Bacterial family percentage in all groups; Fig S1: Analysis of crude polysaccharide from *M. esculenta* mushroom using high-performance liquid chromatography (HPLC).

## Data Availability

The research data generated and/or analysed during the current study are available upon reasonable request from the corresponding author.
