# Supplementary material for: Morchella esculenta polysaccharide attenuate obesity, inflammation and modulate gut microbiota
Source: AMB Express. 2022 Sep 3;12:114. doi: 10.1186/s13568-022-01451-5 (PMC9440975; doi:10.1186/s13568-022-01451-5)
Supplement: Supplementary file 1 — Additional file 1. Table S1: qRTPCR primers sequence; Table S2: Alpha diversity summary; Table S3: Bacterial phylum percentage in all groups; Table S4: Bacterial family percentage in all groups; Fig S1: Analysis of crude polysaccharide from M. esculenta mushroom using high-performance liquid chromatography (HPLC). [file 13568_2022_1451_MOESM1_ESM.docx]

**Supplementary Data**

**Histopathological analysis**

Epididymal adipose and colon tissues of (5mm) thick were fixed in 10% formalin overnight, followed by dehydration, xylene vitrification, and embedded in paraffin. The sections were prepared via microtome (Thermo, Waltham, MA, USA), deparaffinized, rehydrated, and stained with hematoxylin and eosin (H&E). The histological study was performed by a microscope (Leica Microsystems, Wetzlar, Germany) as a blinded independent investigator. The colonic tissue injuries were scored as reported previously. The analysis criteria were as obeys: 0: no significant inflammatory reaction observed; 1: Low-degree of inflammatory reaction observed; 2: Mediumdegree inflammatory infiltration observed; 3: Severe inflammatory reaction observed; 4: High degree of an inflammatory reaction, Cell infiltration observed, and reduced numbers of goblet cells.

**Mucus epithelial thickness and goblet cells analysis**

The mucous layer thickness and goblet cells in the intestine were determined via periodic acid– Schiff (PAS), staining. Tissue sections (5mm) thick were xylene deparaffinized and rehydrated with a grading percentage of ethanol, then treated with periodic acid reagent at room temperature for 5 minutes. The slides were rinsed in water followed by Schiff reagent treatment at room temperature for 7 minutes. Slides were rinsed for 10min under running water and then stain with hematoxylin. The slides were again rinsed with water for 15 minutes, followed by dehydration in ethanol. The slides were mounted and images were captured by a microscope (Leica Microsystems, Germany), the histological study was performed as a blinded independent investigator.

**Immunohistochemistry (IHC) analysis**

To see the expression of mucins protein (MUC2) immunohistochemistry (IHC) staining was performed. The colon tissue sections were deparaffinized, rehydrated, and incubated with 3% H2O2 for 10 minutes. In addition, the tissue slide is heated in an antigen retrieval reagent (Na^+2^ EDTA, pH 8.0) for antigen retrieval. Then incubated overnight with (MUC2) primary antibody at 4 ^◦^C followed by (HRP) horseradish peroxidase-conjugated secondary antibody at room temperature for 1h. The (DAB) 3,3-diaminobenzidine was used as substrate and hematoxylin was applied as counterstaining. The slides were then mounted and examined at a magnification of 20x under a light microscope. under the light microscope at 20x magnification.

**Supplementary tables**

**Table. S1** qRT-PCR primer sequence

| Name | Sequence |
| --- | --- |
| PPAR-γ Forward | 5’-GCAGCTACTGCATGTGATCAAGA-3’ |
| PPAR-γ Reverse | 5’-GTCAGCGGGTGGGACTTTC-3’ |
| FAS Forward | 5’-GCTGCGGAAACTTCAGGAAAT-3’ |
| FAS Reverse | 5’-AGAGACGTGTCACTCCTGGACTT-3’ |
| aFABP Forward | 5’-TGA TGC CTT TGT GGG AAC CT-3’ |
| aFABP Reverse | 5’-GCA AAG CCC ACT CCC ACT T-3’ |
| F4/80 Forward | 5’-TCC AGC ACA TCC AGC CAA AGC-3’ |
| F4/80 Reverse | 5’-CCT CCACTGCATCCAGAAGAAGC-3’ |
| CD68 Forward | 5’-TTCAGGGTGGAAGAAAGGTAAAGC-3’ |
| CD68 Reverse | 5’-CATGATGAGAG GCAGCAAGAGG-3’ |

PPARγ, peroxisome proliferator-activated receptor γ; C/EBPα, ccaat-enhancer-binding protein α;

FAS, fatty acid synthase; aFABP, adipocyte fatty acid binding protein

**Table. S2** Alpha diversity summary

| Groups | Shannon | Simpson | Chao1 | Ace | Goods_coverage |
| --- | --- | --- | --- | --- | --- |
| Control | 6.467 | 0.932 | 1294 | 1260 | 0.9984 |
| Control | 5.644 | 0.897 | 1097 | 1085 | 0.9987 |
| Control | 2.567 | 0.499 | 891 | 881 | 0.9985 |
| FMT | 6.611 | 0.968 | 1457 | 1464 | 0.9979 |
| FMT | 6.623 | 0.970 | 1526 | 1499 | 0.9979 |
| FMT | 6.219 | 0.958 | 1135 | 1132 | 0.9986 |
| HFD | 6.525 | 0.963 | 1293 | 1276 | 0.9987 |
| HFD | 5.678 | 0.945 | 824 | 831 | 0.9990 |
| HFD | 5.678 | 0.945 | 799 | 802 | 0.9990 |
| MEPL | 6.115 | 0.963 | 1075 | 1079 | 0.9985 |
| MEPL | 6.140 | 0.963 | 827 | 831 | 0.9990 |
| MEPL | 6.300 | 0.964 | 1324 | 1321 | 0.9982 |
| MEPH | 4.459 | 0.848 | 759 | 766 | 0.9990 |
| MEPH | 5.912 | 0.947 | 1112 | 1114 | 0.9985 |
| MEPH | 5.674 | 0.901 | 1191 | 1177 | 0.9983 |

**Table. S3** Bacterial phylum percentage in all groups

| Groups | *Firmicutes* | *Actinobacteria* | *Bacteroidetes* | *Proteobacteria* | *TM7* |
| --- | --- | --- | --- | --- | --- |
| Control | 70.16 | 0.79 | 24.84 | 2.01 | 1.44 |
| HFD | 62.37 | 10.50 | 17.09 | 10.02 | 0.06 |
| FMT | 55.93 | 25.41 | 8.46 | 8.39 | 0.36 |
| MEPL | 65.52 | 22.76 | 5.59 | 4.91 | 0.31 |
| MEPH | 55.06 | 23.58 | 12.05 | 6.89 | 1.89 |
|  | ***Deferribacteres*** | ***Tenericutes*** | **Unassigned** | ***Spirochaetes*** | ***Thermi*** |
| Control | 0.18 | 0.84 | 0.02 |  |  |
| HFD | 0.59 | 0.09 | 0.03 | 0.01 | 0.04 |
| FMT | 1.03 | 0.26 | 0.03 | 0.09 | 0.02 |
| MEPL | 0.73 | 0.09 | 0.02 | 0.06 | 0.01 |
| MEPH | 0.03 | 0.38 | 0.04 | 0.02 | 0.02 |

**Table. S4** Bacterial family percentage in all groups

| Groups | *Lactobacillaceae* | *Enterococcaceae* | *Corynebacteriaceae* | *Lachnospiraceae* | *S24-7* | *Ruminococcaceae* |
| --- | --- | --- | --- | --- | --- | --- |
| Control | 53.13 | 0.35 | 0.05 | 14.20 | 15.83 | 6.98 |
| HFD | 4.18 | 25.90 | 6.38 | 13.73 | 13.28 | 14.53 |
| FMT | 13.12 | 5.98 | 28.73 | 8.91 | 5.13 | 6.26 |
| MEPL | 5.96 | 46.89 | 15.53 | 5.81 | 2.91 | 4.83 |
| MEPH | 27.79 | 10.84 | 9.90 | 11.39 | 10.33 | 7.42 |
|  | ***Desulfovibrionaceae*** | ***Nocardiaceae*** | ***Staphylococcaceae*** | ***Rikenellaceae*** | ***Yaniellaceae*** |  |
| Control | 1.58 | 0.00 | 0.16 | 7.46 | 0.26 |  |
| HFD | 8.13 | 6.17 | 1.67 | 4.42 | 1.73 |  |
| FMT | 10.86 | 0.05 | 11.30 | 2.21 | 7.59 |  |
| MEPL | 4.71 | 3.92 | 3.62 | 1.48 | 4.43 |  |
| MEPH | 5.36 | 12.25 | 1.31 | 2.39 | 1.01 |  |

**Supplementary Figures**

**Fig. S1** Analysis of crude polysaccharide from M. esculenta mushroom using high performance liquid chromatography (HPLC).


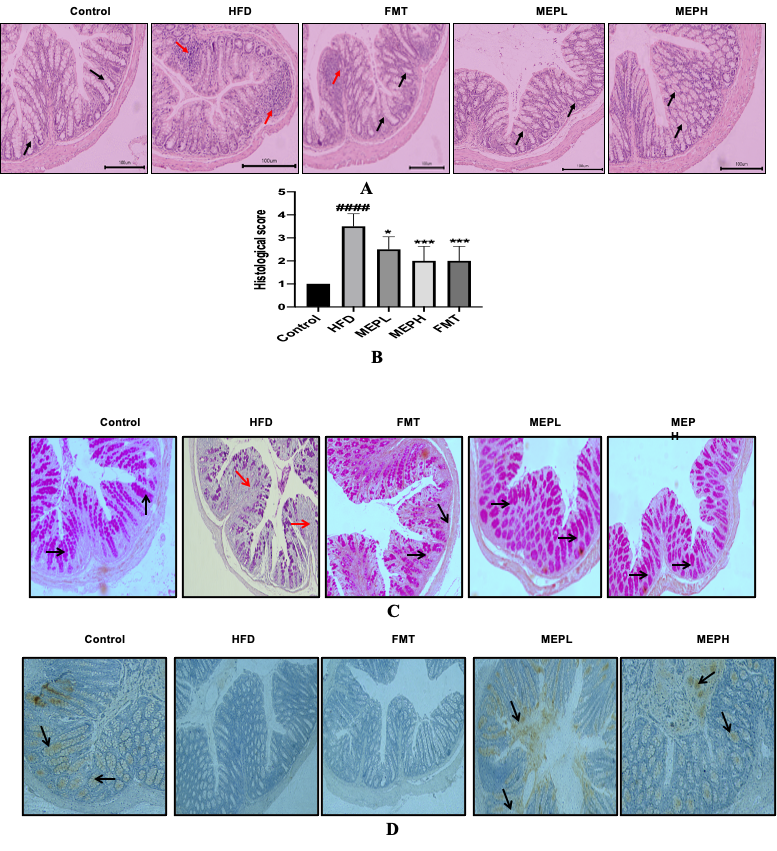


**Fig. S2** MEP supplementation improved histopathological changes and enhanced mucin production in HFD-induced obese model. **A** Photomicrograph of H & E demonstrating the effect of MEP on obesity. The number of goblet cells is represented by black arrows. Red arrows suggest the infiltration of inflammatory cells at magnification 20x. **B** Histological scores or ratings. The data are shown as mean ± SEM.Significant differences were presented using at #p < 0.05, ##p < 0.01 and ###p <0.001 *#*###p <0.0001 *vs.* Control group and *p <0.05, **p < 0.01and *** p < 0.001 **** p < 0.0001 *vs.* HFD group. **C** Photomicrograph of colonic tissue is described by PAS staining. In all groups, the concentration of goblet cells and mucin production were determined. The goblet cells producing mucins are indicated by black arrows. the inflammatory cells infiltration was shown by red arrows at magnification 20x. **D** Photomicrographs of the immunohistochemistry (IHC) of colonic tissue, (black arrow) showed MUC2 expression by the magnification of 20x.


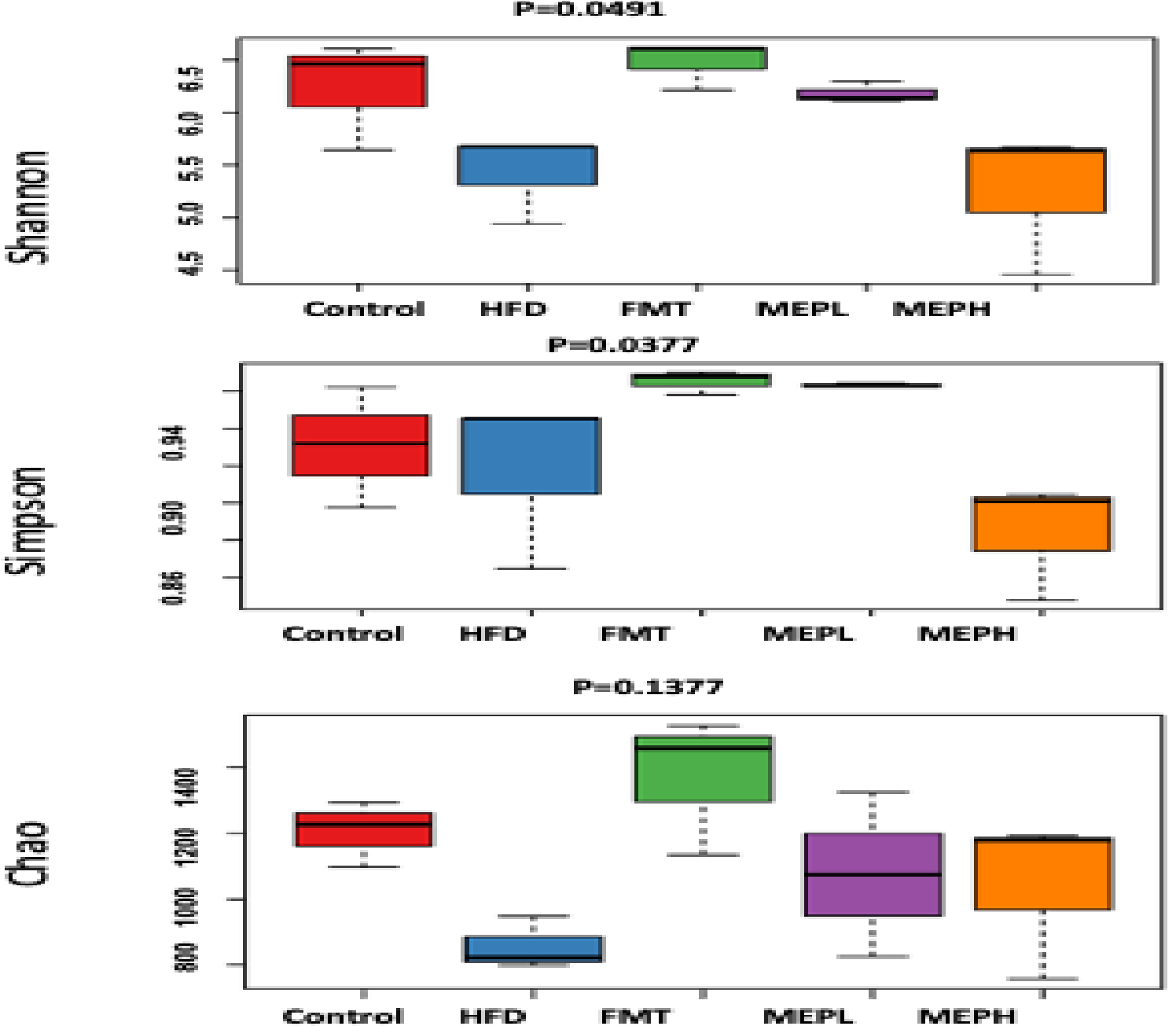


**Fig. S3** Rarefaction measure of Shannon, observed species, and Chao show species diversity, abundance, and evenness.


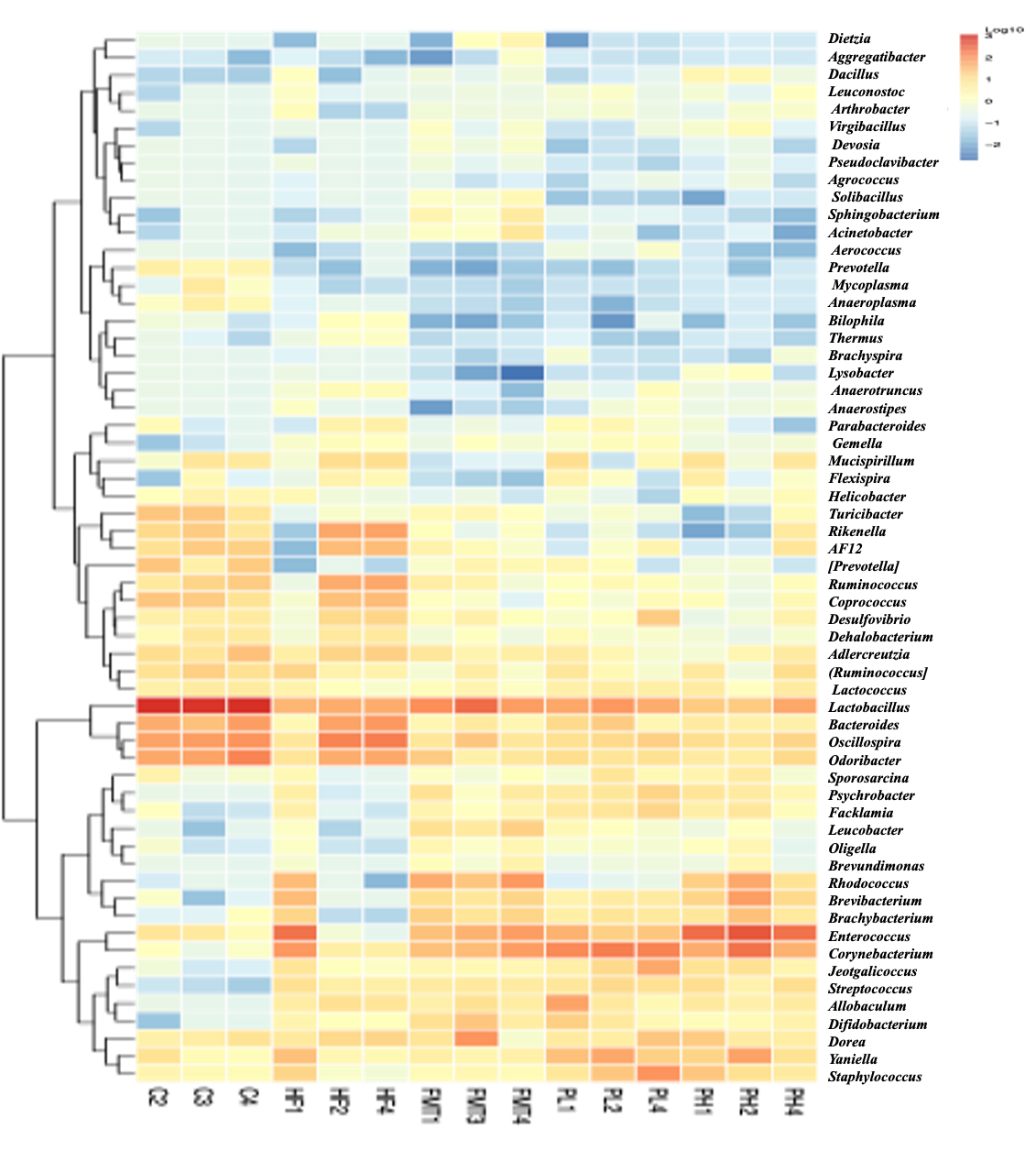


**Fig. S4** Heat map of microbiome composition with clustering analysis. Data of every taxonomic level were clustered following the degree of similarity among Control, HFD, FMT, MEPL, and MEPH groups; the relative abundance of the taxonomic unit and the finding of clustering analysis were arranged individually according to taxonomic units.
